# A Powerful Disinfectant

**Published:** 1880-06

**Authors:** 


### A POWERFUL DISINFECTANT.

Chloride of lead is said to be the most powerful, safe and economical deoderizer and disinfectant known. To prepare it for use, on a small scale, for ordinary purposes, take *half a drachm of the nitrate of lead* and dissolve it in one pint of hot water; dissolve *two drachms common salt* in two gallons of water and mix the solution, this forms a solution of chloride of lead. A cloth wet with this, and hung up in a room filled with fetid atmosphere, will purify it instantly; and the solution thrown into a water-closet, sink or drain, or wherever the sulphite of hydrogen and ammonium exists, or is generated, will produce the same effect. It is not carbonic acid, but the sulphite of hydrogen and ammonium, which are eliminated with the breath, and through the pores of skin, that makes people who are exposed to such an atmosphere so depressed, and which, when highly concentrated, develops typhous poison. Nitrate of lead is in dry crystals and is sold, at about twenty-five cents per pound, one of which would make several hundred gallons of the solution.

PHYSICIAN AND PHARMACIST.

